# Multimodal treatment of a pediatric posterior fossa arteriovenous malformation employing endovascular and microsurgical techniques

**DOI:** 10.3171/2020.10.FOCVID2064

**Published:** 2021-01-01

**Authors:** Zeferino Demartini, Guilherme H. W. Ceccato, Érico S. G. G. da Trindade, Luis A. B. Borba

**Affiliations:** Department of Neurosurgery, Complexo Hospital de Clinicas—UFPR, Curitiba, PR, Brazil

**Keywords:** arteriovenous malformation, intracranial, brain, embolization, child

## Abstract

Intracranial hemorrhage is the most common presentation of posterior fossa arteriovenous malformations (AVMs) and may have serious consequences. The authors present a case of a 7-year-old girl with headache, vomiting, dysmetria, and ataxia due to a ruptured cerebellar grade III AVM. After two sessions of embolization, the patient underwent total microsurgical resection through a suboccipital craniotomy. There were no additional postoperative deficits, and the patient improved progressively during 6 months of rehabilitation. These challenging lesions should be removed after rupture, especially in children with long-term cumulative risk of rebleeding. Multimodal treatment reduces the perioperative bleeding, allowing better outcomes for pediatric AVM.

The video can be found here: https://youtu.be/HQWnjD8ENZQ

## Transcript

**0:21 Introduction.** This video demonstrates the multimodal treatment of a pediatric posterior fossa arteriovenous malformation employing both endovascular and microsurgical techniques.

**0:30 Case Presentation and Exams.** The patient was a 7-year-old female presenting with a history of sudden onset of headache, nausea, and vomiting, associated with dysmetria and gait ataxia. The patient underwent a CT scan that demonstrates two sites of acute bleeding, one inside the upper part of the fourth ventricle and the other at the left petrosal surface of cerebellum.

**0:58** We can better see the sites of bleeding in these coronal and sagittal cuts. The patient developed an obstructive hydrocephalus and an EVD (external ventricular drain) was placed. Investigation of the underlying cause of acute bleeding has proceeded with MRI. In these FLAIR images, we can observe many flow voids in the left cerebellar hemisphere. Here, we see the sequence of 3D-TOF (time of flight) that depicts better the course of vessels in the posterior fossa.

**1:29** Now we can observe the site of bleeding at the petrosal surface of cerebellum, close to the cerebellopontine angle. Magnetic resonance angiography (MRA) depicts a lesion highly suggestive of an arteriovenous malformation (AVM) in the left cerebellar hemisphere, presenting arterial supply suggestive coming from SCA (superior cerebellar artery), AICA (anterior inferior cerebellar artery), and PICA (posterior inferior cerebellar artery), as well demonstrates big arterialized venous channels arising from the nidus of the AVM, and we can better see these features in these coronal cuts.

**1:55** Here we see a 3D reconstruction of the AVM and the arteries from the brain, illustrating its positioning. Now, we can observe the axial cuts from the MRV (magnetic resonance venography), demonstrating engorgement of the left sigmoid sinus and internal jugular vein, and now we have the 3D reconstruction of venous structures demonstrating the dominance of the left side related to the AVM. Here, we see the angiograms performed. A lesion highly suggestive of an AVM was identified, presenting feeders coming from a dilated SCA, as well with filling from AICA and PICA. The nidus measured around 4 cm in diameter. The drainage was both superficial and deep to the transverse and sigmoid sinuses, as well to the basal vein of Rosenthal.

**2:35** We just saw the left vertebral artery injections, and now we can see the ones from the right side. Here, we can observe other images demonstrating these AVM. Considering the size of the nidus and deep venous drainage, the lesion was classified as grade III of Spetzler-Martin and B of Spetzler-Ponce grading systems.

**3:08 Anatomy.** Here, we can review the anatomy related to this case. We can observe the vertebral arteries joining to form the basilar artery and see the SCA, AICA, and PICA, and we see a better relationship with the cranial nerves from a posterior perspective.^1^ We see the SCA, AICA, and PICA.^1^ Here, we have a lateral view demonstrating the left cerebellar arteries arising from the basilar and going around brainstem to reach the cerebellum and feed the AVM.

In this 3D model, we can better observe the configuration of the AVM, presenting feeders coming from all the left cerebellar arteries (SCA, AICA, and PICA), but mainly from a dilated SCA. We observe the nidus and see the dilated veins. Here, we can see the veins draining to the transverse and sigmoid sinuses, as well to the basal vein of Rosenthal that drains into the vein of Galen and then into the straight sinus. We can observe the location of the AVM in the left cerebellar hemisphere, and the anomalous blood vessels circumferentially around the nidus. It is noticed that the left sigmoid sinus and internal jugular vein are significantly dilated compared to the contralateral side and also to the size of internal carotid arteries.

**4:20 Preoperative Embolization.** Considering the complex posterior fossa AVM and that a pediatric patient harbors a lower blood volume, it was chosen to perform preoperative embolization to minimize bleeding during microsurgical resection.^2–6^ Now, we observe the angiograms of the first embolization, employing Onyx. Here, we see a lateral view before embolization, and now observe the first region filled with Onyx, and here the following angiograms. Now, other regions embolized are demonstrated, including apparently some venous channels, suggesting the presence of important AV fistulas.

Here, just a better view, depicting the location of the embolized segments regarding the AVM. Now, we see the angiograms of the second embolization, initially the images prior to Onyx injection, depicting the embolized regions from the previous procedure. Now, we observe the new sites embolized, suggestive of reaching the surface, and here the angiograms performed following it, and we can observe that extensive parts of the AVM are successfully embolized. Here, we can observe the CT scan following the embolization; now we have a 3D reconstruction depicting the Onyx inside the AVM and also a 3D model of the Onyx itself filling the vessels.

**5:48 Microsurgery Planning.** In this case, a suboccipital approach will be employed, exposing at midline but more to the left side.^3,5^ After dural opening, we see the cisterna magna and now observe the cerebellar surface. Then, a partial left cerebellar hemispherectomy will be performed, and here we can observe the approximate location of the AVM regarding this anatomical demonstration.^1^ Here, we can observe the removal of left cerebellar hemisphere in this demonstration, and we can observe the lower cranial nerves running toward the jugular foramen.^1^

**6:20 Positioning and Demonstration of Initial Incision.** The patient was placed in a Concorde position and an L-shaped (“hockey-stick”) incision was performed. A midline suboccipital craniotomy extending more to the left side was employed, and the microsurgical resection was performed with the aid of neurophysiological monitoring.

**6:34 Microsurgical Resection.** Following dural opening, CSF (cerebrospinal fluid) from the cisterna magna is released to achieve brain relaxation. Here, we can observe the lower portion of medulla oblongata, the spinal accessory nerve, and see the PICA with Onyx inside from the preoperative embolization. As this is a big and complex AVM, with huge venous channels inside the cerebellar hemisphere arising from the nidus, in order to decrease the chance of bleeding, it was chosen to not get inside and around the AVM itself and instead to perform an en bloc resection in a partial left cerebellar hemispherectomy, removing the adjacent nonfunctioning gliotic cerebellar tissue.^3,5^

**7:11** Here, we see the inferior lateral edge of resection, exposing the lower cranial nerves. The dissection continues now medially and deep, in order to reach the region anterior to the nidus to attack the arterial blood supply. The dissection progress deeply, and many vessels are found filled with Onyx. After successful coagulation of arterial blood supply, the removal progress coagulating the venous sites of outflow from the AVM to complete the en bloc resection. Now, we inspect the lower cranial nerves entering jugular foramen, and also see the empty cavity with the tentorium pulsating. Here, we observe the spinal root of the accessory nerve, and also see some rootlets of hypoglossal nerve. Also, the initially identified embolized PICA is exposed. Here, it is dissected between the cerebellar hemispheres to expose the floor of the fourth ventricle.

**8:01 Closure of Incision and Postoperative Imaging.** Here, we have a 3D bone reconstruction of the immediate postoperative CT scan, demonstrating the EVD and the suction drain. EVD was placed considering the potential risk of postoperative hydrocephalus. Now, we can observe the axial cuts of the postoperative CT scan, and now the 3D reconstruction.

**8:24 Follow-Up.** The patient presented improvement of headaches, nausea, and vomiting, with no new neurological deficits. She was discharged on postoperative day 7 and underwent rehabilitation for 6 months, presenting gradual improvement of the dysmetria and gait ataxia, identified in the preoperative period, and now we can observe normal gait and no dysmetria following 6 months of rehab.

## Author Contributions

Primary surgeon: Borba. Assistant surgeon: Demartini, Ceccato. Editing and drafting the video and abstract: all authors. Critically revising the work: all authors. Reviewed submitted version of the work: all authors. Approved the final version of the work on behalf of all authors: Demartini. Supervision: Borba.
